# Structural and Functional Analysis of CEX Fractions Collected from a Novel Avastin^®^ Biosimilar Candidate and Its Innovator: A Comparative Study

**DOI:** 10.3390/pharmaceutics14081571

**Published:** 2022-07-28

**Authors:** Busra Gurel, Melike Berksoz, Eda Capkin, Ayhan Parlar, Meltem Corbacioglu Pala, Aylin Ozkan, Yılmaz Capan, Duygu Emine Daglikoca, Meral Yuce

**Affiliations:** 1 SUNUM Nanotechnology Research and Application Center, Sabanci University, Istanbul 34956, Turkey; busra.gurel@sabanciuniv.edu; 2 ILKO ARGEM Biotechnology R&D Center, Istanbul 34906, Turkey; mberksoz@sabanciuniv.edu (M.B.); edacapkin@sabanciuniv.edu (E.C.); mcorbacioglu@ilkogen.com.tr (M.C.P.); aozkan@ilkogen.com.tr (A.O.); ycapan@ilko.com.tr (Y.C.); 3 Faculty of Engineering and Natural Sciences, Sabanci University, Istanbul 34956, Turkey; ayhanparlar@sabanciuniv.edu

**Keywords:** biosimilars, monoclonal antibodies, avastin, bevacizumab, cation exchange chromatography, charge variants, fractionation, mass spectrometry, surface plasmon resonance

## Abstract

Avastin^®^ is a humanized recombinant monoclonal antibody used to treat cancer by targeting VEGF-A to inhibit angiogenesis. SIMAB054, an Avastin^®^ biosimilar candidate developed in this study, showed a different charge variant profile than its innovator. Thus, it is fractionated into acidic, main, and basic isoforms and collected physically by Cation Exchange Chromatography (CEX) for a comprehensive structural and functional analysis. The innovator product, fractionated into the same species and collected by the same method, is used as a reference for comparative analysis. Ultra-Performance Liquid Chromatography (UPLC) ESI-QToF was used to analyze the modifications leading to charge heterogeneities at intact protein and peptide levels. The C-terminal lysine clipping and glycosylation profiles of the samples were monitored by intact mAb analysis. The post-translational modifications, including oxidation, deamidation, and N-terminal pyroglutamic acid formation, were determined by peptide mapping analysis in the selected signal peptides. The relative binding affinities of the fractionated charge isoforms against the antigen, VEGF-A, and the neonatal receptor, FcRn, were revealed by Surface Plasmon Resonance (SPR) studies. The results show that all CEX fractions from the innovator product and the SIMAB054 shared the same structural variants, albeit in different ratios. Common glycoforms and post-translational modifications were the same, but at different percentages for some samples. The dissimilarities were mostly originating from the presence of extra C-term Lysin residues, which are prone to enzymatic degradation in the body, and thus they were previously assessed as clinically irrelevant. Another critical finding was the presence of different glyco proteoforms in different charge species, such as increased galactosylation in the acidic and afucosylation in the basic species. SPR characterization of the isolated charge variants further confirmed that basic species found in the CEX analyses of the biosimilar candidate were also present in the innovator product, although at lower amounts. The charge variants’ in vitro antigen- and neonatal receptor-binding activities varied amongst the samples, which could be further investigated in vivo with a larger sample set to reveal the impact on the pharmacokinetics of drug candidates. Minor structural differences may explain antigen-binding differences in the isolated charge variants, which is a key parameter in a comparability exercise. Consequently, such a biosimilar candidate may not comply with high regulatory standards unless the binding differences observed are justified and demonstrated not to have any clinical impact.

## 1. Introduction

Monoclonal antibodies (mAbs) as therapeutic drugs have gained significant attention due to their specific and sensitive treatment potential for some cancer types, and autoimmune or neurodegenerative diseases [1,2,3]. The biosimilar mAb candidates are expected to have an analytical, functional, and clinical similarity with the innovator molecule for approval [4,5,6,7]. The similarity acceptance criteria are outlined and regulated by authorities such as the FDA, EMA, ICH, and WHO, which require additional, comprehensive analyses in case of deviations from the predefined, analytical biosimilarity range [7,8,9]. The manufacturers have to establish critical quality attributes (CQAs) to ensure consistent product efficacy, safety, and quality [10,11]. CQAs involve multiple analyses to reveal the biosimilar candidate’s physical, chemical, and biological similarities or dissimilarities to the innovator product.

Monitoring charge variant similarity throughout the development of mAbs is one of the critical quality requirements [12,13,14]. Recombinant mAb products may contain heterogeneous charge variants, usually resulting from post-translational modifications occurring during cell culture, formulation, and storage. The post-translational modifications that occur enzymatically or non-enzymatically during the upstream and downstream drug development processes can cause charge heterogeneity [15,16]. Charge variants are composed of acidic, main, and basic species. Some modifications, including cyclization of N-terminal glutamines, clipping of C-terminal lysine, or glycation, lead to modifications in the protein’s net charge. Other modifications such as methionine oxidation or aspartic acid isomerization generate the charge variants by affecting the local charge distribution [17,18]. The charge heterogeneities may or may not affect the biological activity of the mAbs despite the chemical and structural differences resulting from the modifications [19]. For example, two commercial Avastin^®^ biosimilars approved by EMA and FDA, Mvasi [20,21] and Zirabev [22], showed microcharge heterogeneity. It is known that C-terminal lysine and N-terminal pyro-Glu can generate specific charge isoforms, but it was reported that such variations had no significant impact on the in vitro potency, effector function, or pharmacokinetics of some mAbs [19,23]. On the other hand, modifications or other physicochemical differences in the Antigen-Binding Fragment (Fab), Complementary Determining Region (CDR), or the Fragment Crystallizable region (Fc) can alter the binding ability of a mAb to its target antigen or receptor proteins [24], in addition to their possible impact on drug absorption and bioavailability [25]. For instance, Complement-Dependent Cytotoxicity (CDC) was reduced by removing galactose residue, while Antibody-Dependent Cellular Cytotoxicity (ADCC) activity was enhanced by removing fucose residues [26]. The aggregation, fragmentation, or misfolding caused by the host cell line or the process itself may also lead to the loss of biological activity or several other side effects [27]. Thus, the chemical and functional characterization of unexpected charge heterogeneity becomes critical for quality assessment.

Chromatographic methods, specifically cation and anion exchange chromatography techniques (CEX, AEX), are extensively used to characterize the charge-based heterogeneities of mAbs [28]. In CEX, the acidic variants are eluted earlier, and the basic variants are eluted later than the main peak. After determining the acidic, main, and basic variant distribution via CEX analysis, fractionation is performed for the in-depth characterization of each charge isoform [29,30,31]. Fraction-based methods provide a more sensitive determination of modifications by eliminating the interfering proteoforms arising from the cross-contamination of different charge species. The fraction collection can be conducted manually or automated, and further analysis can be performed with the fractions of interest following the proper buffer exchange, if necessary [24,32]. The in-depth characterization of the separated charge variants has gained significant attention to understand better the different species’ physicochemical roots in innovator and biosimilar products [33].

Avastin^®^ is a recombinant humanized immunoglobulin type 1 (IgG1) monoclonal antibody that targets vascular endothelial growth factor type A (VEGF-A) to inhibit angiogenesis in various cancer types. It prevents the progress of tumors by binding and neutralizing all VEGF-A isoforms [34,35]. SIMAB054 is an Avastin^®^ biosimilar candidate in the flask-scale development stage, showing significant microcharge heterogeneity, especially in basic charge variants, compared to the innovator. In this study, the fractionated charge variants of SIMAB054 were characterized in detail by comparing them to the fractionated charge variants of Avastin^®^. Following the fractionation of both products’ acidic, main, and basic variants, all samples were analyzed by UPLC-MS at the intact protein and peptide levels to investigate the post-translational modifications and major glycoforms causing charge dissimilarity. Furthermore, the VEGF-A and Neonatal receptor (FcRn)-binding capacities of the samples were investigated in vitro by Surface Plasmon Resonance (SPR) assays to demonstrate the potential differences in these functional parameters. The results shed light on each variant’s structural differences and receptor- and antigen-binding capacities, which were not discussed in the previous reports and EMA/FDA documents.

## 2. Material and Methods

### 2.1. Cation-Exchange Chromatography (CEX)

Avastin^®^ (AVT/AVT08, Genentech, San Francisco, CA, USA, Lot: 33808339) and SIMAB054 (produced by ILKO ARGEM Biotechnology R&D Center, Istanbul, Turkey) samples were diluted to 9.4 mg/mL with ultrapure distilled water and injected into the HPLC directly. CEX separation was performed on BioPro SPF Non-porous Column (5 µm, 100 × 4.6 mm, YMC). Mobile phase A:100 mM NaH_2_PO_4_·2H_2_O (Merck), mobile phase B:100 mM Na_2_HPO_4_·2H_2_O (Merck, Kenilworth, NJ, USA), and mobile phase C:1 M NaCl (Merck) were prepared with mass spectrometry (MS) grade water. Auto Blend method was used to perform a salt gradient from 0 mM to 200 mM sodium phosphate at constant pH of 5.7. The flow rate was 0.5 μL/min, and the column temperature was 25 °C. Detection was achieved using a PDA detector (Waters, ACQUITY PDA Detector) at 280 and 214 nm wavelengths. Raw data were processed by EMPOWER Software. The acidic and basic variants of AVT and SIMAB054 in CEX analysis were collected by fraction manager equipment in the HPLC system. Samples were loaded to HPLC at high concentration to get high yield fractions. The method was adapted from Ref. [36].

### 2.2. Size-Exclusion Chromatography (SEC)

AVT and SIMAB054 samples were diluted to 1 mg/mL with pure deionized water, and a 20 µL sample was injected into the HPLC system (Waters, ACQUITY HPLC-PDA Detector, Markham, ON, USA). SEC analysis was performed using TSK-GEL G3000SWxL (7.8 × 300 mm, TOSOH, Tokyo, Japan) column. Mobile phases made of 0.2 mol/L potassium phosphate and 0.25 mol/L NaCl pH 6.2 were prepared with MS-grade water. The flow rate of the isocratic method was 0.33 uL/min, and the column temperature was 25 °C. Detection was conducted by a PDA detector at 280 nm wavelengths. The raw data were processed by EMPOWER Software (Empower 3, Waters, Milford, MA, USA).

### 2.3. Intact Protein Analysis

AVT08 and SIMAB054 samples were diluted to 0.5 mg/mL with 50 mM ammonium bicarbonate (AMBIC, Sigma-Aldrich, St. Louis, MO, USA) and injected directly into the LC-MS/MS system (Waters, ACQUITY UPLC-ESI-Xevo G2-XS QToF). Mobile phase A was MS grade water (Merck), mobile phase B was ACN (Merck), and mobile phase C was 1% FA (Merck). The reverse-phase separation was performed on ACQUITY UPLC-BEH300 C4 1.7 µm column (2.1 mm × 50 mm, Waters) using a 1 min gradient (5–85% B). During the run, the flow rate and column temperatures were 0.4 μL/min and 80 °C. The mass range was set to 500–4000 *m*/*z* and analyzed in ESI-positive and sensitivity mode. The instrument was calibrated using NaCsI (Sigma-Aldrich), and Glu-1-fibrinopeptide B (Waters) was used as a lock-mass reference. The method was adapted from reference [37].

The deconvolution of raw mass spectra of intact mAb samples was performed by the UNIFI MaxEnt1 algorithm (Waters) with the following parameters: input *m*/*z* range, 2400–3200; output mass range, 146,500–150,000; minimum intensity ratio left and right, 30%, FWHM, 0.73 (low *m*/*z*) and 0.92 (high *m*/*z*); the number of iterations, 20. Major glycoforms (G0F, G1F, G2F, G0) and C-terminal lysine were introduced as modifications, and only the components identified with <50 ppm mass error were accepted as glycoforms. The percentage of each glycoform was calculated using the formula: “Response % Glycoform = (Response/Total Response of Glycoforms) × 100” [38].

### 2.4. Peptide Mapping Analysis

AVT and SIMAB054 samples (50 ug) were treated with 1% SDS (Sigma-Aldrich) and 0.1 M DTT (Sigma-Aldrich) in 50 mM AMBIC solution and incubated at 56 °C for 15 min. After the reduction, samples were alkylated with 20 mM IAA (Sigma-Aldrich) for 30 min in the dark at room temperature. After incubation, all samples were diluted with 8 M urea and purified with 30 kDa MWCO disposable filter units (Millipore) at 14,000 g for 20 min. The purified samples were incubated with 1 ug trypsin (Pierce) in 75 μL AMBIC (1:50, *w*/*w*, enzyme to protein ratio) at 37 °C overnight. The filtrates, including tryptic peptides, were collected by washing the filter unit with 50 uL of 50 mM AMBIC twice. Finally, the samples were acidified with 1% formic acid before analysis [39].

The tryptic peptides were analyzed by ACQUITY UPLC-ESI-Xevo G2-XS QToF system (Waters). Mobile phase A comprised MS grade water, mobile phase B was ACN, and mobile phase C was 1% FA. The percentage of mobile C was set to 10%, and the percentage of mobile phase B was increased from 1 to 80% over an 85 min total run time. The instrument was calibrated with NaCsI, and Glu-1-fibrinopeptide B (100 fmol/uL) was used as a lock-mass reference. Data-independent acquisition mode (DIA) was performed by sequential MS, and MS/MS scans with 0.5 s cycle time. The mass range was set to 50–2000 *m*/*z* and all ions within the range were fragmented without any precursor ion selection in sensitivity mode.

The raw data were processed with UNIFI by applying peptide mapping workflow parameters. The reference sequence was retrieved from http://www.drugbank.ca/ (accessed on 1 February 2022). Trypsin was selected as a digesting reagent with one missed cleavage maximum. Carbomidomethyl-C was set as a fixed modification because of the alkylation step in the sample preparation, while the other modifications (Oxidation-M, deamidation-N, succinimide intermediates, pyroglutamic Acid-N term) were set as a variable. The mass tolerance window was set within 10 ppm. The components with greater than 10% matched primary ions (b/y ions), <10 ppm mass error, and no in-source fragment were allowed for identification. The percentage of modifications was calculated using the following equation: “%peptide = (Response of modified peptide/Total response of the modified and unmodified peptides) × 100” [38,40].

### 2.5. VEGF-Binding Assays

The VEGF-binding analyses were performed on VEGF 165A-immobilized (Sigma-Aldrich) CM5 chips with a Biacore T200 SPR Instrument (Cytiva) [41]. The chip surface was prepared with standard EDC-NHS coupling chemistry [42]. VEGF165A was diluted to 5 ng/µL in pH 5.5 in 10 mM acetate buffer. Following the activation of surface carboxylate groups by EDC/NHS injection, the target protein, VEGF 165A, was covalently immobilized through the free primary amine groups. The excess number of activated carboxyl groups on the matrix was blocked with a 1 M ethanolamine-HCl (Cytiva) injection. The final immobilization level for the active flow cell reached approximately 500 response units (RU) for all experiments. An ethanolamine-immobilized flow channel was used as the control surface. The CEX-fractionated charge variant samples at three concentrations (15 nM, 5 nM, 1.66 nM) were prepared in 1X HBS-EP buffer (10 mM HEPES, 150 mM NaCl, 3 mM EDTA, 0.005% *v*/*v* polysorbate 20) at pH 7.4, which was also employed as the running buffer. Single-cycle kinetic analyses were conducted at 30 µL/min flow rate at 22 °C. Analytes were injected for 120 s in the association phase, followed by a dissociation phase of 1800 s with the running buffer. Blank measurements were also performed on the active and control flow channels by running buffer injections under identical conditions. The chip surface was regenerated by injecting 10 mM glycine pH 1.5 buffer for 90 s. Results were obtained by subtracting responses from blank flow cells and zero concentration analyte injection (running buffer). Two innovator samples (AVT08 and ALT03) were used in all SPR experiments of the unfractionated, initial samples. The SPR data were presented as the mean value, calculated from at least five measurements per sample. The equilibrium dissociation constants (K_D_) were calculated by Biacore Evaluation Software using the 1:1 Langmuir binding model [42]. An equivalence test was used for evaluation of the similarity of SIMAB054 charge variants to innovator.

### 2.6. FcRn-Binding Assays

FcRn-binding analyses of the fractionated samples were carried out on Anti-His IgG1 antibody (Cytiva) immobilized CM5 chips (Cytiva). Anti-His IgG1 antibody immobilization procedure was applied by an amine coupling kit based on the manufacturer’s guide (Cytiva). His-Tagged FcRn (Sigma-Aldrich) protein and all other charge variants or whole molecule samples were prepared in 1X HBS-EP pH 6.0 running buffer with three-fold dilutions (15 nM, 5 nM, 1.66 nM). The recombinant His-tagged FcRn molecule was captured on the active flow cell for 120 s with a 10 µL/min flow rate at 22 °C. A blocked flow cell was used as a blank during all measurements. Samples were injected over both flow cells (active and blank) at a 30 µL/min flow rate for 120 s, followed by the dissociation phase of 900 s with the running buffer. The chip surface was regenerated with 1X HBS-EP buffer (pH 7.4) for 60 s. Blank buffer injections were also performed on both flow channels, which were later subtracted from the active surface data before the fitting. Two innovator samples (AVT08 and ALT03) were used in all SPR experiments of the unfractionated, initial samples. The SPR data were presented as the mean value, calculated from at least 5 measurements per sample. One-way analysis of variance, ANOVA, was used to reveal the statistically significant differences between the fractionated sample pairs (*p* < 0.05 was considered significant and *p* < 0.005 was considered highly significant). The results were evaluated with Biacore Evaluation Software using the steady-state [43,44] and two-state binding models [45,46].

## 3. Results and Discussion

### 3.1. CEX and SEC Analyses of Fractionated Charge Variants

Charge heterogeneity is one of the most common types observed in mAbs and should be considered part of CQAs. Each quality attribute is assigned to one of the three risk tiers recommended by the FDA [47], and CEX is categorized as one of the Tier 2 attributes with its moderate clinical effect [48]. Despite its mild effect, a comprehensive characterization of the charge isoforms is necessary for the initial risk assessment. In addition, detailed information about the biochemical roots and possible biological impacts of any undesired charge variant must be presented to the authorities [49]. Among several chromatographic and electrophoretic methods available for charge variant analysis [50,51,52], CEX is a widely used, qualitative technique with high resolution [14,34,35,53,54]. SIMAB054 is an Avastin^®^ biosimilar candidate in the flask-scale development stage, showing significant microcharge heterogeneity, especially in basic charge variants, compared to the innovator. Here, we physically separated all charge variants of SIMAB054 and the innovator product (AVT) using the CEX method to investigate each variant’s structural and functional behavior in detail.

**Figure 1:**
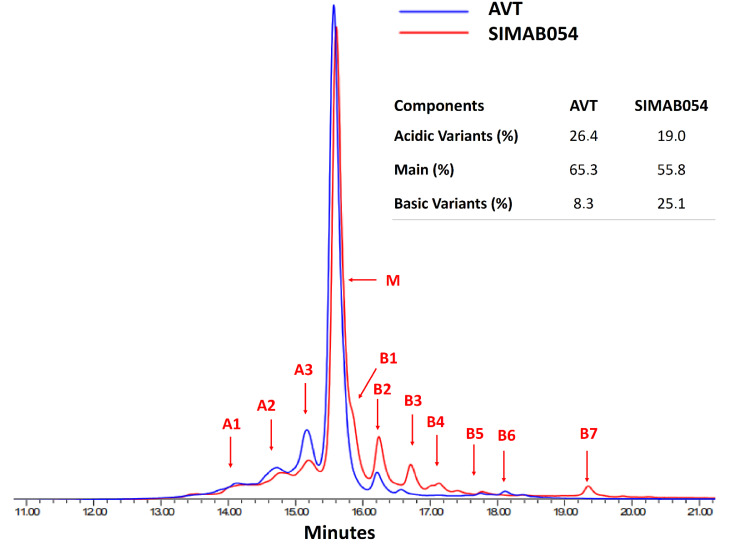
CEX results of AVT and SIMAB054. Overlay of CEX results of AVT and SIMAB054 and a comparative table of acidic, basic, and main charge variant percentages.

The distribution of the acidic, main, and basic variants of both products were represented as an overlay CEX chromatogram, and the average percentages of each indicated variant were given as an inset table in Figure 1. Acidic variants were approximately 26% for AVT and 19% for SIMAB054, while the basic variant percentages of SIMAB054 (25%) were significantly more than AVT (8%). Additionally, several basic peaks were observed in SIMAB054, but not in AVT. Peak fractionation was designed as shown in Figure 1; three acidic (A1–A3), seven basic (B1–B7) variants, and the main variants were obtained for each sample.

**Figure 2:**
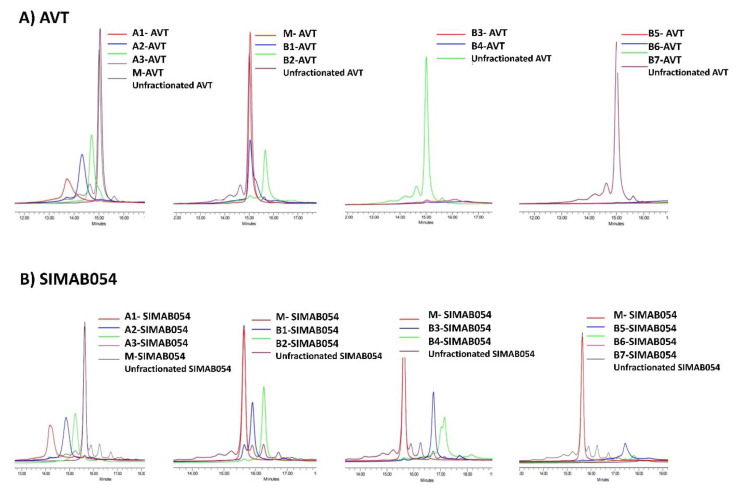
Each fraction was analyzed by CEX to confirm the presence of targeted charge variants. (**A**) CEX fractions collected from the innovator, AVT. (**B**) CEX fractions collected from SIMAB054.

**Figure 3:**
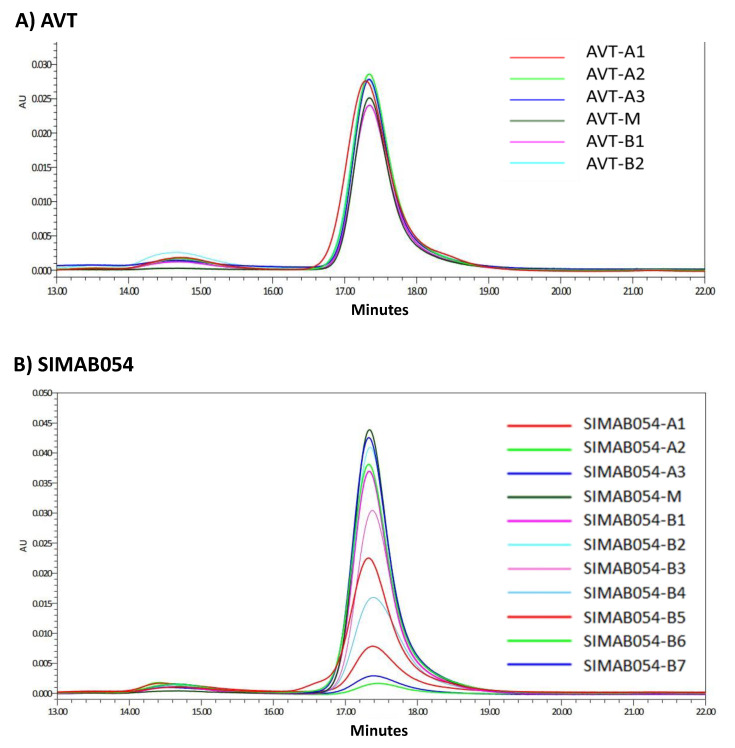
SEC-HPLC analysis results of each fraction. (**A**) Overlay chromatograms of AVT08 charge variant fractions. (**B**) Overlay chromatograms of SIMAB054 charge variant fractions.

The purity of the separated fractions was verified by the re-analysis of each fraction via CEX (Figure 2). Each charge variant was physically separated by a fraction collector following the CEX analysis. In order to elucidate the physicochemical roots of each charge variant in AVT and SIMAB054, several types of analytical tools were used. One was size exclusion chromatography (SEC) which provides information about the molecular size, hydrodynamic radius, and the apparent molecular mass [55]. SEC analyses of the fractionated samples revealed the monomeric content of the samples (Figure 3). Some samples were not detectable by the SEC due to the insufficient sample concentration. The SEC analyses showed that each fraction of both products contained similar size species. The monomer peak was resolved between the 17th and 18th minute. A high molecular weight (HMW) peak was observed between the 14th and 15th minute for both products. None of the fractions contained low molecular weight (LMW) species.

### 3.2. Intact Protein Analysis of Fractionated Charge Variants

The chemical characterization of the charge variant fractions was initially performed by intact protein analysis using mass spectrometry. Intact protein analysis is one of the most critical structural elucidation tools for mAbs. The intact mAb analysis reveals the exact molecular weight of the sample. The method also helps to define the modifications that cause a significant molecular weight shift (>100 kDa), such as glycosylations (>1200 Da) and C-terminal lysine clippings (128 Da) [56]. As one of the best-studied modifications, glycosylation is found in the highly conserved Asn303 residue in each heavy chain of Avastin^®^ [57], and its potential correlation with charge heterogeneities was previously reported [58]. It is already known that the major N-linked glycoform of Avastin^®^ is a biantennary fucosylated and agalactosylated structure (G0F: G0F) with fully clipped C-terminal lysine residues (molecular mass: ~149.2 kDa) [57]. The SIMAB054 showed the same dominant glycoform when analyzed in its initial form, without charge variant fractionation. All charge variant fractions of the AVT and SIMAB054 were analyzed by intact analysis under the same experimental conditions to determine whether there were any glycoform differences between the charge species. The B5, B6, and B7 basic fractions were pooled together before the analysis to increase the sample concentration. The final sample was labeled as B5 for all remaining analyses.

**Figure 4:**
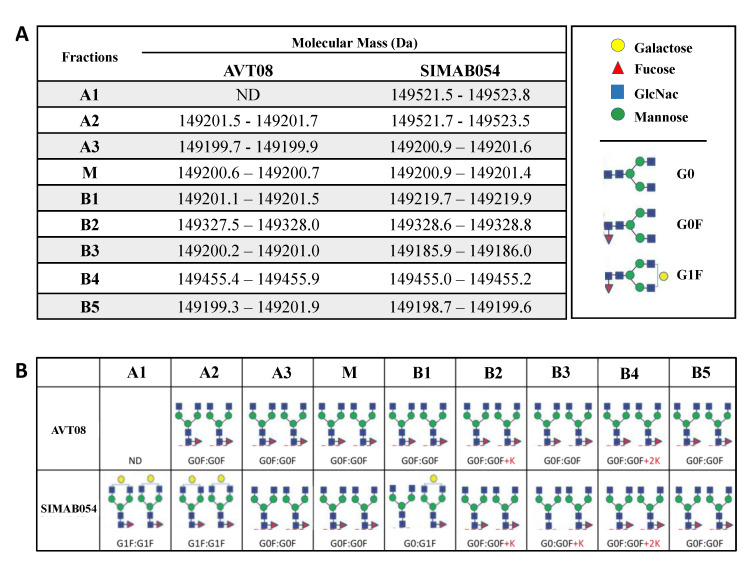
Intact protein analysis of charge variant fractions obtained from AVT and SIMAB054. (**A**) The list of the molecular mass of the dominant mass peak in each fraction. Each fraction was injected three times, and the mass ranges represent the minimum–maximum observed mass values. A, M, and B represent acidic, main, and basic fractions. (**B**) Illustration of glycoforms assigned for each molecular mass.

The molecular mass of the main peak in the deconvoluted MS spectrum of each fraction was listed in Figure 4A. All fractions contained one dominant mass peak identified by matching the observed and expected theoretical mass with a ±50 ppm mass error. Figure 4B represents the glycoforms and modifications assigned to the dominant mass. Similar to the unfractionated AVT and SIMAB054 samples, both the products’ main and A3 fractions have G0F:G0F with clipped C-term lysine (149,200 Da). In the AVT, G0F: G0F was also assigned to the A2 fraction, whereas no dominant glycoform was detectable for the A1 sample. A mass difference of 320 Da was observed in A1 and A2 fractions of SIMAB054, identified as monogalactosylation (G1F:G1F) on both chains (149,520 Da). The results are consistent with the study, which demonstrates that the terminal galactosylation content of acidic charge variants was higher than the main fraction, and the levels of the acidic variants were linearly increased by the increased G1F content [41]. In the basic variants, incomplete C-terminal lysine clipping was identified as the predominant modification rather than the glycoforms, although there were some exceptions. It is known that differences in the levels of C-terminal lysine residues of monoclonal antibodies administered by the intravenous route are not expected to impact product performance, as it is typically removed in vivo shortly after administration [20,21,22].

In detail, the B1 peak (149,219 Da), observed only in SIMAB054 in CEX, was identified as an afucosylated and galactosylated (G0:G1F) form. It is known that afucosylation leads to basic variants [24], while the terminal galactose can lead to acidic variants, as mentioned before. Therefore, both modifications in the same molecule could be why B1 appeared as a shoulder to the main peak. In the B2 fractions of AVT and SIMAB054, an unclipped lysine residue at one heavy chain (G0F:G0F + K) was identified for the observed mass (149,328 Da) because it is known that lysin leads to a +128 Da mass shift. Similar to B2, two Lys residues (G0F:G0F + 2K) were identified in the B4 fractions of both AVT and SIMAB054, with a + 456 Da mass difference (149,456 Da). Unlike the AVT, the B3 fraction of SIMAB054 also had one lysine residue in the C-terminal, in addition to afucosylation at one heavy chain (G0:G0F + K, 149,186 Da). Finally, the B5 fraction was similar in AVT and SIMAB054 samples regarding glycoforms and C-terminal clipping (G0F:G0F, 149,200 Da). The incomplete lysine truncation formed during the upstream process is a frequent reason for basic species formation [59,60,61]. It is known that the lysine residues are naturally cleaved off in circulation due to the intrinsic carboxypeptidase activity of the cells [18]. Lysin removal has no known effect on antibody function or structure, mostly related to C1q activity and CDC [62,63]. This experiment’s findings may not be evaluated as a direct reason for the charge heterogeneity, but it may contribute to the literature by revealing various glycoproteoforms in different charge species. The dominant glycoforms in the fractions were identified, and two significant findings were figured out, despite the limitations in the analysis method. First, the partially or non-clipped C-terminal lysine was dominant in basic charge variants. Secondly, terminal galactosylation was observed mainly in acidic species, while afucosylation was detected in the basic species.

### 3.3. Peptide Mapping Analysis of Fractionated Charge Variants

Several enzymatic or non-enzymatic modifications such as oxidation, deamidation, glycation, or N-terminal pyroglutamic acid have been reported to form acidic or basic charge variants [31,64]. Although intact (MS) analysis is useful for analyzing several modifications, it is hard to assign chemical modifications with small mass differences. Therefore, post-translational modifications (PTMs) such as deamidation (+1 Da), oxidation (+16 Da), and N-terminal cyclization (−17 Da) were analyzed by the data-independent acquisition LC-MS^E^ method to reveal the differences beyond the charge variants of AVT and SIMAB054. Table 1 represents the percentages of indicated PTMs in selected peptides of each fraction. The percentage values were calculated by averaging three separate injections of the pooled samples.

Several reports have shown that IgG oxidation is mainly related to serum half-life [65,66]. The antigen-binding capacity is usually not affected by oxidation because the most susceptible residues for oxidation are found in the CH2 domain [62]. Oxidation usually occurs in methionine and appears in tryptophan, histidine, and other residues at a lesser amount. Although Met252 and Met428 (according to EU numbering [67]) display a higher susceptibility towards oxidation, additional Met residues can be oxidized due to different forced studies [68,69]. As seen in Table 1, the methionine oxidation of HC/T19 (DTLM_ox_ISR) containing Met252 is significantly higher (>10%) in basic fractions than in main and acidic peaks, while the oxidation of LC/T1(DIQM_ox_TQSPSSLSASVGDR) remains the same (~1%) beyond all fractions of AVT and SIMAB054. The oxidation of all remaining methionine and tryptophan residues was calculated under 2% in all fractions, such as LC/T1. This result is not surprising because oxidation-M is a known modification leading to basic species formation [64]. It is also known that some peptides are prone to modifications because of the structural conformation of the mAbs [70].

Asparagine (Asn) deamidation and aspartate (Asp) isomerization are other extensively studied modifications as significant factors of the chemical degradation of mAbs unless the appropriate storage and formulation conditions were provided [71,72]. Additionally, prior studies have noted the importance of the Asn/Asp site because the deamidation of Asn residues in the antigen-binding sites can lead to a loss of potency or functionality of mAbs [73]. Deamidation is a non-enzymatic reaction and occurs by the hydrolysis of the amide group, which causes a +1 Da molecular mass shift, which cannot be easily detected by intact protein analysis [74]. Therefore, the deamidation level was analyzed in each charge species by peptide mapping analysis. In several peptides, an extreme deamidation level (>25%) was observed in almost every fraction, probably enhanced by the sample preparation step of peptide mapping, including reduction, alkylation, and tryptic digestion [75]. Table 1 represents the deamidation level of selected peptides that have a reasonable deamidation level. No significant differences in the Asn deamidation level of LC/T9 (SGTASVVCLLN_deam_NFYPR) and HC/T8 (STAYLQMN_deam_SLR) were observed throughout charge species of the same groups. In contrast, notable differences (two- or three-fold) were found in the deamidation level of HC/T21 (FNWYVDGVEVHNdeamAK) in acidic and basic SIMAB054 compared to the AVT. This finding is unexpected because it is known that Asn deamidation leads to the formation of acidic species [60,76]. There might be other dominant modifications affecting the antibody’s net charge. The level of isomerization and succinimide, an intermediate product of asparagine deamidation, appeared to be unchanged throughout the fractions of both AVT and SIMAB054.

The cyclization of the N-terminal is formed rapidly in blood circulation following the mAb administration [77] by the chemical or enzymatic conversion of glutamine (Gln) or glutamic acid (Glu) at the N-terminals of the mAbs to pyroglutamate (pyroGlu) [78]. The N-terminal of the Avastin’s heavy chains has Glu residue, which is cyclized to pyroGlu by removing an H_2_O molecule. Therefore, the presence of pyroGlu does not affect the charge heterogeneity of the Avastin^®^, but enhances the molecule’s hydrophobicity, which makes this modification easily detectable by reverse-phase chromatography. Different groups previously reported that the pyroGlu could be found in acidic or basic species [28,29,79]. In our study, the N-terminal cyclization of HC/T1 (_pyro_EVQLVESGGGLVQPGGSLR) is found relatively higher (two-to-three-fold) in acidic species of AVT than in the basics. The highest pyroGlu ratio was found in the A3 fraction of both AVT (~6%) and SIMAB054 (~3%), which may be accepted as a minor modification. Although no study shows the benefit of pyroGlu, formed by an enzymatic reaction or in a pH-dependent manner in vivo or in vitro, it is evaluated by the biopharmaceutical industry as a part of process control [80].

According to the peptide mapping of charge species, AVT and SIMAB054 have similar increasing methionine oxidation levels in basic species. It is relatively lesser in SIMAB054 than AVT. Asn deamidation is surprisingly increased in basic species of SIMAB054, which can be explained by considering the effect of other dominant modifications. N-terminal pyroGlu levels in all charge species of AVT and SIMAB054 are similar and reach the highest amount in the A3 fraction. As with methionine oxidation, the pyroGlu level is relatively lesser in SIMAB054 than in AVT. These results also suggest that all charge variant fractions contain almost all kinds of PTMs with variable percentages, which can be assumed to be a reason for the related species’ enhanced amount.

### 3.4. VEGF-Binding Analysis of Fractionated Charge Variants

Avastin^®^ is an IgG1-type antibody that neutralizes the circulating VEGF molecules by binding them with the Fab regions. Neutralizing the VEGF molecule prevents its interaction with target receptors (VEGFR) on the endothelial cells [81]. The binding kinetics of a mAb to its target molecule is known to be affected by charge heterogeneities formed during manufacturing and other processes, and other parameters such as stability and effector function [82,83,84]. An Enzyme-Linked Immunosorbent Assay (ELISA) [85], Biolayer interferometry (BLI) [41,86], KinExA [87], and SPR-based methods [88,89] are commonly utilized to investigate antibody–antigen interactions. The SPR technique is robust and reliable for characterizing the binding events, label-free in real-time [90]. In this study, SPR was used to reveal the binding affinities of the different charge variants of AVT08 and SIMAB054 against the VEGF and FcRn molecules. The binding kinetics of the initial molecules without fractionation was also investigated.

**Figure 5:**
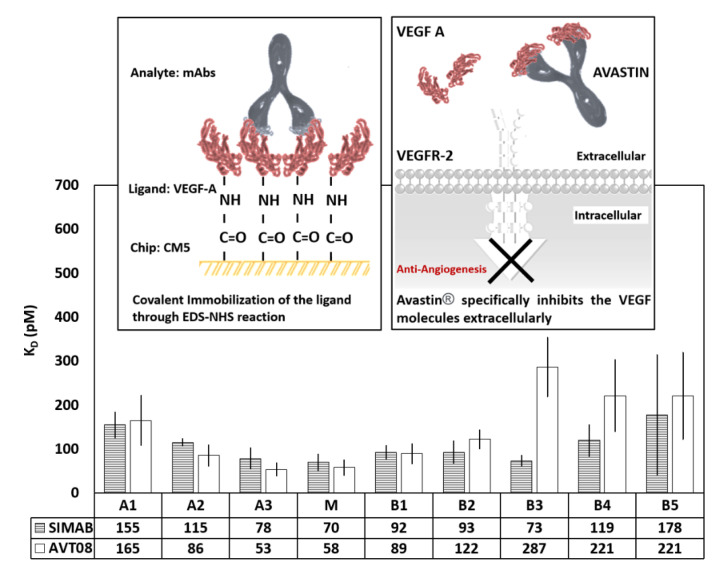
The VEGF-binding characteristics of the fractionated charge variants using the SPR using the Langmuir 1:1 binding model. The samples fractionated by the CEX method were obtained from the innovator (AVT) and the biosimilar candidate (SIMAB054) under the same operational conditions. A, M, and B, respectively, represent acidic, main, and basic fractions. The K_D_ data were presented as the mean value obtained from at least five measurements. The inset (top-left) is a representative illustration of the prepared SPR chip surface. The inset (top-right) shows the proposed mechanism of action for Avastin^®^, adapted from reference [91].

SPR-based VEGF-binding analysis of the fractionated charge variants was presented in Figure 5. According to the data, the M fractions from both products showed a similar trend with the highest VEGF affinity among all samples tested. It was not surprising that A3 and B1 variants showed the second-best binding values since they were located right next to the M variant in the CEX analysis and behaved similarly for VEGF binding. The lowest binding performance was found in the B4 and B5 fractions with the highest standard deviation, probably due to a lower purity. Among the acidic fractions, the A2 sample from the AVT performed slightly better (86 ± 24 pM) than the A2 fraction obtained from the SIMAB054 (115 ± 8 pM) product. The A1 samples from both products presented a similar binding ability towards the targeted VEGF antigen, calculated as 155 ± 26 and 165 ± 56 pM for SIMAB054 and AVT fractions, respectively. The difference between the B3 fractions from the reference and the innovator product was significant. The binding constant values increased from B2 to B5 for both products, in which the B3 fractions from the SIMAB054 product presented a better binding ability (lower K_D_ values) in every case. The minimum and the maximum K_D_ values for these basic species were recorded as 73 ± 11–221 ± 81 pM, respectively.

**Figure 6:**
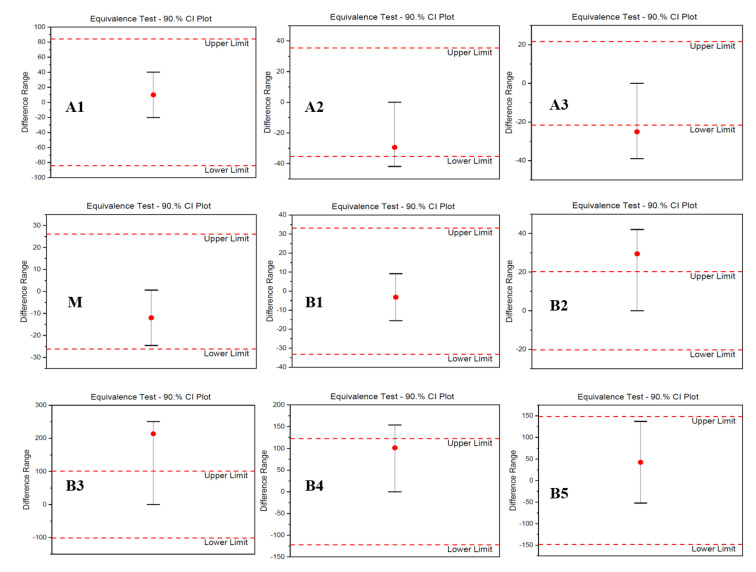
The VEGF-binding characteristics of the fractionated charge variants using the SPR using the Langmuir 1:1 binding model. The samples fractionated by the CEX method were obtained from the innovator (AVT) and the biosimilar candidate (SIMAB054) under the same operational conditions. A, M, and B, respectively, represent acidic, main, and basic fractions. The means of K_D_ values were obtained from at least five measurements, and an equivalence test was used to compare each charge variant of SIMAB054 with those of AVT.

Two-sample equivalence test was used to show the means difference between the samples in a 90% confidence interval. The equivalence test results for SPR-based VEGF-binding analysis of the fractionated charge variants are presented in Figure 6. The main fractions from both products showed a similar trend with the highest VEGF affinities among all samples tested. According to the equivalence test, A1, M, B1, and B5 variants were found statistically not different and equivalent to the innovator, while A2 and B4 variants were found not different, but also not equivalent. On the other hand, A3, B2, and B3 variants seemed not equivalent and statistically different from the same variants of the innovator.

According to the peptide map results, those basic fractions mostly contained unclipped Lysine residues at a greater ratio in SIMAB054 than in the AVT fractions. It was reported in the literature that the unclipped Lysine residues do not usually change the biological function of the product [19,24], and the endogenous carboxypeptidase B activity of the cells degrades the extra Lysine residues immediately [92]. For example, the basic charge isoforms of an IgG antibody (with 15% leader sequence and 85% C-terminal Lys) presented similar potency and pharmacokinetics in rats [93]. On the other hand, the major glycoforms varied slightly among the tested fractions, and the oxidation levels of the basic species between two products’ basic fractions were observed at different ratios (found less in the SIMAB). Such variations in the defined modifications may have caused the altered antigen-binding behavior of the basic species. Du et al. [94] reported that the modifications in CDR could lead to the formation of extra acidic (such as deamidation of Asn) or basic (Asp isomerization, succinimide formation, and Met oxidation) variants, and such acidic variants had lower binding activities towards the target molecules due to the conformational changes.

Several other examples in the literature correlate the modifications with the biological functions similarly, but for different mAb samples. Vlasak et al. reported that acidic fractions of the antibody that contained deamidation in the Fab region had a reduced binding activity to the target molecule [95]. Another study presented that the formation of the deamidation intermediate in CDR2 (Asn55) reduced the Ka compared to the native Fab form [96]. Moreover, the characterization study of Trastuzumab indicated that the acidic fraction of the antibody had a lower binding affinity than the main and basic fraction of the antibody to the HER2 [97]. As reported in the literature, modifications in CDR may alter the antigen affinity; however, no significant modification was observed in the CDR of either product. Thus, we could not directly correlate the basic species’ increased VEGF-binding capabilities with the defined modifications. Such a direct correlation may require a detailed force degradation study supported by SPR and proliferation assays that might be a future project.

On the other hand, it was noted in the SPR data that the SD values for the basic species B3-B5 were significantly higher than the other fractions, for example, 137 pM for the SIMAB054-B5 fraction and 99 pM for the AVT-B5 fraction (data not shown), probably due to the physicochemical diversity of the IgG1 molecules in these samples that may not have separated precisely enough during the CEX fractionation. The initial separation range had been intentionally kept wider for the basic species to collect the samples at a beneficial concentration, which did not work for the fractions with low percentages. The number of the basic species separated from the CEX was more significant at the beginning of the study; later, the B5, B6, and B7 were pooled to increase the final sample concentration. Although there were differences between the calculated K_D_ values, especially the basic species, the values were still in the VEGF-binding range reported previously by other groups [19,88,98]. The K_D_ differences for different charge variants of the same mAb sample were also previously reported for other mAb samples [7]. Despite the importance of charge heterogeneity in biosimilars, there are limited studies investigating charge variant-specific binding kinetics. The present study offered a side-by-side SPR characterization of the fractionated innovator and a biosimilar candidate.

### 3.5. FcRn-Binding Analysis of Fractionated Charge Variants

The FcRn is a cell surface receptor expressed on monocyte and endothelial cells. This receptor prevents IgG degradation in endosomes and extends the IgG molecules’ half-life in vivo by binding to the Fc parts of IgG molecules in a pH-dependent manner [99]. Post-translational modifications on mAbs can affect their interaction with the receptor proteins. For example, methionine oxidation reduced the mAb’s interaction with the FcRn receptor by leading to conformational changes in the structure [45,100,101]. On the other hand, deamidation was shown to increase the affinity of the mAbs to the FcRn [93]. In order to examine the interaction of antibodies with the Fc receptors, an amplified luminescent proximity homogenous assay (Alpha screen) [102,103], ELISA, and Fluorescence Resonance Energy Transfer-based assays (FRET) [104] were proposed in the literature. Although the in vivo studies are the most useful in observing the biological impact of different charge variants or modifications on pharmacokinetics, it is not possible for every research group. Thus, real-time in vitro analysis by SPR can be a robust alternative to the previous methods for FcRn-binding characterizations. In the current study, all isolated charge isoforms of AVT08 and SIMAB054 products were investigated by the SPR against the captured FcRn ligands to reveal the binding patterns among different samples.

The FcRn-binding affinity of two different lots of the unfractionated initial innovator samples (AVT08 and ALT03) and the SIMAB054 were 25.03 ± 7.63, 22.72 ± 1.69, and 22.02 ± 1.86 nM, respectively (data not shown in a graphical format). The binding affinity of Avastin^®^ to human recombinant FcRn molecules was previously reported in a range from 6 to 2500 nM with different techniques [44,69,105]. In an SPR-based FcRn–IgG interaction study, the affinity values were reported between 6.58 ± 0.12 and 49.6 ± 1.78 nM for recombinant IgG and between 9.99 ± 0.43 and 71.9 ± 15.7 nM for human IgG1 [106]. The FcRn-binding interaction of mAbs by SPR is considered in the Tier 2 category, with a low risk on the product quality [107]. The analytical biosimilarity range for this interaction was represented as the mean ± 3×SD [107], calculated from at least five measurements of the two innovator products (AVT08, ALT03), obtained from two different lots. Under the specified conditions, the analytical biosimilarity range for the FcRn-mAb interaction was calculated as 7.66–40.09 nM, which qualifies the initial, unfractionated SIMAB054 product as a potential Avastin^®^ biosimilar candidate in the FcRn-binding parameter.

**Figure 7:**
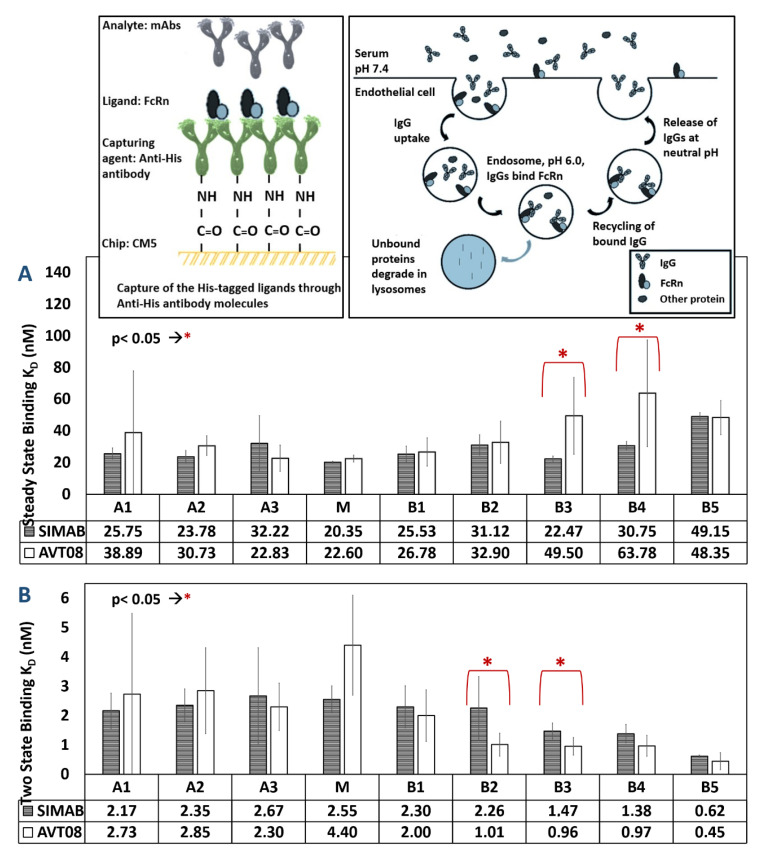
The FcRn-binding characteristics of the fractionated charge variants were revealed by the SPR, using steady-state (**A**) and two-state binding models (**B**). The samples fractionated by the CEX method were obtained from the innovator (AVT08) and the biosimilar candidate (SIMAB054) under the same operational conditions. The A, M, and B represent acidic, main, and basic fractions. A lower K_D_ value represents a better binding. The K_D_ data were given as the mean value obtained from at least five measurements. The inset (top-left) is a representative illustration of the prepared SPR chip surface. The inset (top-right) shows the proposed mechanism of action for FcRn, adapted from reference [108].

The comparative steady-state FcRn-binding [43,44] data of the fractionated charge variants were presented in Figure 7A. The data were also analyzed with two-state binding kinetics [45,46] to reveal possible differences between the applied fitting models presented in Figure 7B. The main fractions of both AVT08 and SIMAB054 gave the highest binding affinity towards the captured FcRn ligands, with calculated K_D_ values of 22.60 ± 2.21 nM and 20.35 ± 0.62 nM for AVT08 and SIMAB054, respectively. According to the ANOVA analysis, the acidic species obtained from both products, A1, A2, and A3, presented a similar FcRn-binding affinity that remained in the range of 22.83 ± 2.28 to 38.89 ± 38.91. Among the basic species, the B3 and B4 samples presented a statistically significant difference (*p* < 0.05, 95% confidence interval), while the variants of SIMAB054 presented a better binding affinity towards FcRn than the same variants from the AVT08 product. The K_D_ values for the B3 fractions from the AVT08 and SIMAB054 products were 49.50 ± 24.19 and 22.47 ± 1.73, respectively.

On the other hand, the KD values for the B4 fractions from AVT08 and SIMAB054 products were calculated as 63.78 ± 33.59 and 30.75 ± 2.70, respectively. Finally, the B1, B2, and B5 isoforms presented a statistically similar binding trend towards the FcRn in both products, with a calculated K_D_ between 25.53 ± 5.01 and 49.15 ± 2.38 nM. Two-state binding kinetics analysis of the same data gave almost similar results, except for the B2 variants, which were found statistically different (*p* < 0.05) between the innovator and the SIMAB054 samples.

The literature has no general agreement about the correlation between the charge heterogeneity and the FcRn-binding ability. The findings reported in some studies indicated an increased affinity for FcRn, especially the basic variants [24,109], and some other studies showed that the basic variants of a recombinant antibody bound better to the FcRn receptor than the acidic variants, which contained sialylation, glycation, and deamidation modifications [41,93,109]. According to a different study, there was no significant difference between the IgG molecule’s basic and acidic variants regarding the FcRn-binding ability [41]. In the current data set, the acidic species generally showed a similar binding affinity for FcRn compared to the M variant and the initial, unfractionated products. The late basic species (B3, B4, and B5) presented slightly lower binding affinities, which were still within the standard deviations’ limits compared to the other samples and the calculated analytical biosimilarity range (7.66–40.09 nM). The overall data suggest no significant FcRn-binding affinity change among the tested charged isoforms obtained from the innovator and biosimilar candidate molecules under the same experimental conditions. Howevre, the K_D_ values changed in between the main, acidic, and basic species. It should be noted that the SPR data alone is not enough to predict in vivo antibody stability or the serum half-life. Khawli et al. reported that the administration of separated antibody fractions (acidic, main, and basic) into the rats did not affect the pharmacokinetic features, although the minor differences in K_D_ values beyond the charge variant fractions were observed by SPR [93].

The overall evaluation and summary of the results obtained by chemical and biological activity characterization studies are represented in Table 2. The distribution of each charge species in CEX was expressed as the peak percentages. The dominant glycoforms and the significant variations in modifications were summarized for both AVT and SIMAB054 isoforms.

## 4. Conclusions

SIMAB054 is an Avastin^®^ biosimilar candidate in the flask-scale development stage, showing significant microcharge heterogeneity, especially in basic charge variants compared to the innovator. The current study’s scope was to reveal the structural and functional behaviors of the physically separated charge variants from SIMAB054 and the innovator product in a comparative manner. A side-by-side characterization approach was applied to the samples using CEX, UPLC-MS/MS, and SPR techniques. The CEX study revealed that some basic species in the innovator product do not form detectable peaks during the analysis, which can be easily misinterpreted as the absence of such species in the innovator product. However, the charge variants’ isolation from the innovator and the biosimilar candidate under the same conditions showed that the innovator product actually contained similar basic charge variants, although at lower amounts. The SPR characterization of the isolated charge variants further confirmed that basic species found in the CEX analyses of the biosimilar candidate were also present in the innovator product, although at lower amounts. Another critical finding was the presence of different glyco proteoforms in different charge species, such as increased galactosylation in the acidic species and afucosylation in the basic species. The same analysis confirmed that the incomplete C-terminal Lysine clipping led to the formation of basic variants. Methionine oxidation, asparagine deamidation, and N-terminal cyclization were also analyzed as potential factors affecting the VEGF- and FcRn-binding affinities, and several minor differences among the charge variants were observed.

The minor or major alterations in the chemical structure do not always significantly affect the biological activity of a monoclonal antibody. However, there were significant deviations from the reference in the antigen-binding data obtained from the SIMAB054 charge variant isoforms despite the biosimilarity found in its intact or unfractionated protein form. Minor structural differences in this study may explain antigen-binding differences in the isolated charge variants, which is a key parameter in a comparability exercise. Further characterization of the isolated charge variants can be performed using various analytical tools such as Circular Dichroism, Dynamic Light Scattering, Fourier Transform Infrared spectroscopy, and Hydrogen/Deuterium Exchange Mass Spectroscopy. The isolation of the charge variants from degraded (under oxidative, UV, heat, or other conditions) or enzymatically-treated products can also be used to investigate the impact of a specific stress condition or modification in vitro. Consequently, such a biosimilar candidate may not comply with high regulatory standards unless the binding differences observed are justified and demonstrated not to have any clinical impact.

## Figures and Tables

**Table 1:** The post-translational modification alterations of selected peptides in all charge variant fractions. The results were obtained by peptide mapping analysis of AVT and SIMAB054. The percentage values were calculated by averaging three separate injections, indicating standard deviations. A, M, and B represent acidic, main, and basic fractions. HC, heavy chain; LC, light chain; TX, tryptic peptide number in the indicated chain.

Charge Variant	Sample Type	Oxidation M		Deamidation N			Isomerization	Succinimide N		N-Term Cyclization
		LC/T1	HC/T19	LC/T9	HC/T8	HC/T21	LC/T9	HC/T2	HC/T34	HC/T1
**A1**	**AVT08**	1.01 ± 0.07	9.86± 0.56	6.21 ± 0.42	3.49 ± 0.04	3.04 ± 0.06	12.47 ± 0.94	0.94 ± 0.00	1.00 ± 0.03	2.48 ± 0.22
	**SIMAB054**	1.22 ± 0.03	8.94 ± 0.26	5.82 ± 0.29	3.29 ± 0.2	5.86 ± **0.00**	11.92 ± 0.72	0.91 ± 0.00	0.91 ± 0.02	1.08 ± 0.08
**A2**	**AVT08**	1.43 ± 0.09	8.35 ± 0.62	6.24 ± 0.34	4.37 ± 0.3	4.84 ± 1.44	12.50 ± 0.78	0.95 ± 0.02	0.85 ± 0.02	2.48 ± 0.17
	**SIMAB054**	1.07 ± 0.04	7.49 ± 0.29	5.83 ± 0.31	3.38 ± 0.1	2.61 ± 0.00	11.28 ± 0.73	0.94 ± 0.00	0.85 ± 0.01	1.17± 0.04
**A3**	**AVT08**	0.92 ± 0.04	6.11 ± 0.48	5.65 ± 0.33	2.81 ± 0.05	2.61 ± 0.04	11.39 ± 0.75	0.95± 0.04	0.84 ± 0.02	6.18 ± **0.45**
	**SIMAB054**	1.24 ± 0.04	7.03 ± 0.5	4.90 ± 0.39	2.49 ± 0.2	4.22 ± **0.00**	9.93 ± 0.72	0.96 ± 0.06	0.86 ± 0.02	3.20 ± **0.16**
**M**	**AVT08**	ND	4.15 ± 0.33	5.75 ± 0.32	2.66 ± 0.07	3.53 ± 1.56	11.48 ± 0.64	0.98 ± 0.01	0.84 ± 0.01	0.91 ± 0.09
	**SIMAB054**	0.83 ± 0.00	3.02 ± 0.15	5.07 ± 0.3	2.35 ± 0.1	4.65 ± 0.00	10.30 ± 0.69	0.97 ± 0.02	0.82 ± 0.08	0.71 ± 0.06
**B1**	**AVT08**	1.86 ±0.02	10.26 ±0.95	5.83 ± 0.3	2.90 ± 0.03	2.65 ± 0.00	11.85 ± 0.75	0.94 ± 0.03	0.86 ± 0.01	1.39 ± 0.07
	**SIMAB054**	1.02 ± 0.01	11.03 ± **0.78**	5.90 ± 0.44	3.45 ± 0.25	5.62 ± **0.00**	11.97 ± 0.89	0.90 ± 0.01	0.86 ± 0.02	1.14 ± 0.03
**B2**	**AVT08**	1.31 ± 0.04	10.11 ± **0.44**	5.78 ± 0.39	2.87 ± 0.07	2.48 ± 0.04	11.60 ± 0.78	0.93 ± 0.01	0.87 ± 0.02	1.10 ± 0.05
	**SIMAB054**	1.10 ± 0.02	7.70 ± 0.17	6.15 ± 0.45	3.21 ± 0.07	6.00 ± **0.00**	12.44 ± 0.83	0.88 ± 0.02	0.85 ± 0.02	0.88± 0.04
**B3**	**AVT08**	1.26 ± 0.05	15.52 ± **0.75**	5.89 ± 0.5	3.09 ± 0.09	2.61 ± 0.1	11.87 ± 1	0.93± 0.04	0.85 ± 0.02	1.67 ± 0.1
	**SIMAB054**	1.17 ± 0.07	10.35 ± **0.2**	6.26 ± 0.4	2.85 ± 0.15	4.04 ± **1.86**	12.61 ± 0.7	0.95 ± 0.03	0.88 ± 0.01	1.05± 0.04
**B4**	**AVT08**	1.36 ± 0.03	16.45 ± **0.4**	5.81 ± 0.4	3.12 ± 0.16	3.49 ± 1.36	11.74 ± 0.87	0.94 ± 0.02	0.86 ± 0.02	1.58 ± 0.12
	**SIMAB054**	1.01 ± 0.09	11.11 ± **0.3**	5.90 ± 0.5	2.95 ± 0.15	ND	11.81 ± 1.1	0.97 ± 0.01	0.94 ± 0.01	0.91± 0.04
**B5**	**AVT08**	1.30 ± 0.12	18.70 ± **0.57**	3.86 ± 0.3	1.75 ± 0.08	3.53 ± 0.00	7.67 ± 0.62	0.81± 0.00	1.10 ± 0.02	1.51 ± 0.12
	**SIMAB054**	0.93 ± 0.00	16.26 ± **0.56**	6.12 ± 0.7	2.82 ± 0.13	2.76 ± 0.00	12.21 ±1.25	0.90 ± 0.01	1.09± 0.04	0.93 ± 0.06

**Table 2:** A summary of the results obtained by chemical and biological activity characterization studies was represented.

	CEX Peak (%)	AVT	SIMAB054	Result
**A1**	AVT: 4.71% SIMAB054: 3.78%	Glycoforms could not be characterized. No dominant modification was observed.	Predominant glycoform is G1F: G1F with truncated Lys. A slight increase in deamidation.	✓ Glycoforms could not be compared. ✓ Similar PTM profiles. ✓ Similar in VEGF binding. ✓ Similar in FcRn binding.
**A2**	AVT: 8.24% SIMAB054: 6.96	Predominant glycoform is G0F: G0F with truncated Lys. No dominant modification was observed.	Predominant glycoform is G1F: G1F with truncated Lys. No dominant modification was observed.	✓ Difference in predominant glycoform. ✓ Similar PTM profiles. ✓ Different in VEGF binding (*p* < 0.05). ✓ Similar in FcRn binding.
**A3**	AVT: 12.24% SIMAB054: 7.5%	Predominant glycoform is G0F: G0F with truncated Lys. Increase in deamidation and N-terminal cyclization.	Predominant glycoform is G0F: G0F with truncated Lys. Increase in deamidation and N-terminal cyclization.	✓ No difference in predominant glycoform. ✓ Both contain PyroGlu at N-term. ✓ Different in VEGF binding. ✓ Similar in FcRn binding.
**M**	AVT: 65.30% SIMAB054: 55.8%	Predominant glycoform is G0F: G0F with truncated Lys.	Predominant glycoform is G0F: G0F with truncated Lys.	✓ No difference in predominant glycoform. ✓ Similar PTM profiles. ✓ Similar in VEGF binding. ✓ Similar in FcRn binding.
**B1**	AVT: NA% SIMAB054: 7.61%	Predominant glycoform is G0F: G0F with truncated Lys.	Predominant glycoform is G0: G1F with truncated Lys.	✓ Difference in predominant glycoform. ✓ Similar PTM profiles. ✓ Similar in VEGF binding. ✓ Similar in FcRn binding
**B2**	AVT: 3.52% SIMAB054: 8.17%	Predominant glycoform is G0F: G0F with 1 Lys. Increased in oxidation and deamidation.	Predominant glycoform is G0F: G0F with 1 Lys. Increased in oxidation and deamidation.	✓ No difference in predominant glycoform. ✓ Same PTMs with variable ratios. ✓ Similar in VEGF binding. ✓ Similar in FcRn binding.
**B3**	AVT: 1.56% SIMAB054: 3.89%	Predominant glycoform is G0F: G0F with truncated Lys. Increased in oxidation and deamidation.	Predominant glycoform is G0: G0F with 1 Lys. Increased in oxidation and deamidation.	✓ The difference in predominant glycoform. ✓ Same PTMs with variable ratios. ✓ Different in VEGF binding (*p* < 0.005). ✓ Different in FcRn binding (*p* < 0.05).
**B4**	AVT: 0.91% SIMAB054: 1.57%	Predominant glycoform is G0F: G0F with 2 Lys. Increased in oxidation.	Predominant glycoform is G0F: G0F with 2 Lys. Increased in oxidation.	✓ No difference in predominant glycoform. ✓ Same PTMs with variable ratios. ✓ Different in VEGF binding (*p* < 0.05). ✓ Different in FcRn binding (*p* < 0.05).
**B5**	AVT: 1.35% SIMAB054: 1.96%	Predominant glycoform is G0F: G0F with truncated Lys. Increase in oxidation, deamidation, and isomerization.	Predominant glycoform is G0F: G0F with truncated Lys. Increase in oxidation, deamidation, and isomerization.	✓ No difference in predominant glycoform. ✓ Same PTMs with variable ratios. ✓ Similar in VEGF binding. ✓ Similar in FcRn binding.

## Abbreviations

DTT	1,4-Dithiothreitol
IAA	iodoacetamide
AMBIC	ammonium bicarbonate
SDS	sodium dodecyl sulfate
ACN	acetonitrile
NaCsI	sodium cesium iodide
FDA	U.S. Food and Drug Administration
ICH	International Council for Harmonization
EMA	European Medicines Agency
WHO	World Health Organization
CQA	critical quality attributes
AVT	Avastin^®^
FcRn	neonatal Fc receptor
VEGF	vascular endothelial growth factor
K_D_	dissociation constant
